# A qualitative study exploring barriers to adequate uptake of antenatal care in pre-conflict Syria: low cost interventions are needed to address disparities in antenatal care

**DOI:** 10.1186/s40834-021-00156-7

**Published:** 2021-06-01

**Authors:** Rima Mourtada, Hyam Bashour, Fiona Houben

**Affiliations:** 1 Department of Epidemiology and Population Health, London School of Hygiene and Tropical Medicine, Keppel St, Bloomsbury, London, WC1E 7HT UK; 2 Faculty of Health Sciences, American University of Beirut, Bliss Street, Beirut, Lebanon; 3 Faculty of Medicine, University of Damascus, Damascus, Syria; 4 Faculty of Health and Wellbeing, Canterbury Christ Church University, North Holmes Road, Canterbury, Kent, CT1 1QU UK

**Keywords:** Antenatal care, Conflict, Interventions, Syria

## Abstract

**Background:**

Syria has made progress in reducing maternal mortality and morbidity before the conflict in 2011. Despite the improvement in antenatal care (ANC) coverage and patterns of use, analyses of national surveys demonstrated wide regional variations in uptake, timing and number of visits even after controlling for women’s socio-demographic characteristics. This study compares two governorates: Latakia, where uptake of ANC was high and Aleppo, where uptake of ANC was low to highlight the barriers to women’s adequate uptake of ANC that existed in Syria pre-conflict.

**Methods:**

This qualitative study carried out 30 semi-structured interviews with (18–45-year-old) pregnant women from Aleppo and Latakia (recruited purposively from different types of health facilities in rural and urban areas), and 15 observation sessions at health facilities. Transcripts and fieldnotes were analyzed using the Framework Method with attention to the dimensions of availability, accessibility and acceptability of services.

**Results:**

Inadequate uptake of ANC in Aleppo included not attending ANC, seeking care with providers who are not trained to provide ANC or discontinuing care. Three themes explained the regional disparities in the uptake of ANC in Aleppo and Latakia: women’s assessment of their health status and reasoning of causes of ill health in pregnancy; women’s evaluation of the risks of seeking ANC; and women’s appraisal of the value of different types of service providers. Poor experiences at public health facilities were reported by women in Aleppo but not by women in Latakia. Evaluations of ANC services were connected with the availability, accessibility (geographical and financial) and acceptability of ANC services, however, women’s views were shaped by the knowledge and prevailing opinions in their families and community.

**Conclusions:**

Findings are utilized to discuss low-cost interventions addressing the disparities in ANC uptake. Interventions should aim to enable vulnerable women to make informed decisions focusing on regions of low uptake. Women’s groups that foster education and empowerment, which have been effective in other low resource settings, could be of value in Syria. Increased use of mobile phones and social media platforms suggests mobile health technologies (mHealth) may present efficient platforms to deliver these interventions.

**Supplementary Information:**

The online version contains supplementary material available at 10.1186/s40834-021-00156-7.

## Background

The vast majority of pregnancy-related complications that cause maternal and neonatal morbidity and mortality take place in low and middle-income countries (LMICs) [1, 2]. Most interventions, often introduced during antenatal care (ANC), improve the health of the mothers and the neonates concurrently [3].

ANC is defined as the care provided by skilled health-care providers to pregnant women and adolescent girls in order to ensure the best health conditions for both mother and baby during pregnancy [4]. It is recommended that ANC should be initiated in the first trimester of pregnancy because most interventions should be implemented early in order to be effective [5, 6]. A minimum number of visits is important. The World Health Organization (WHO) formerly recommended four focused ANC visits for low-risk pregnancies with additional visits for high risk women following the 1990 model [7], however, this model was found to be associated with higher perinatal deaths compared to the model advocating for 8 visits [8]. Consequently, WHO’s updated model in 2016 recommended 8 ANC visits; one visit in the first trimester, 2 visits in the second trimester and 5 visits in the third trimester [8].

Given the prominent role of ANC in protecting the health of mothers and babies, understanding the factors that influence women’s access to and uptake of ANC services is extremely important in order to address any barriers that prevent demand for or provision of such services. A number of health seeking behaviour models have been developed to map some factors that have shown to have an influence on patients’ health seeking behaviour. The most commonly used models, which focus on utilisation of the health services are the Pathway Models, the Health Care Utilisation Model or the Andersen Model and the “Four As” Model [9].

The Pathways Models describe the sequence of events that patients follow starting with acknowledging the symptoms, and ending with the use of different types of health services (traditional and non-traditional) [10]. Good’s model, one example of the Pathway Models, highlights the crucial role of family and friends in the management of illness and consequently the decision making process regarding different therapeutic choices [11].

The Andersen and Newman Health Care Utilisation Model [12, 13] groups in a logic sequence three categories of factors, which influence health behaviour or treatment actions: 1- Predisposing factors that include the socio-cultural characteristics of the individual that existed prior to their health condition such as age, education, parity, health awareness and previous use of the service; 2- enabling factors that include the means or logistics required to obtain the services, and 3- needs factors that include perception of health status of the individual and perception of usefulness of health services.

The “Four As” model assembles key factors for health-seeking behaviour into four dimensions of access that overlap with “the Right to Health” elements as well. “The Right to Health means that governments must generate conditions in which everyone can be as healthy as possible**.”** Such conditions range from ensuring availability of health services, healthy and safe working conditions, adequate housing and nutritious food [14]. The “Right to Health” contains four elements: 1-availability: a sufficient quantity of functioning public health and health care facilities, goods and services, as well as programmes, 2-accessibility: health facilities, goods and services accessible to everyone and it has four overlapping dimensions (non-discrimination, physical accessibility, economical accessibility (affordability) and information accessibility). 3- acceptability: all health facilities, goods and services must be respectful of medical ethics and culturally appropriate as well as sensitive to gender and life-cycle requirements. 4- quality: health facilities, goods and services must be scientifically and medically appropriate and of good quality [15].

The health services “access” concept is also a function of demand and supply. Demand-side factors are defined as “those factors that affect demand and that operate at the individual, household or community level” whereas supply side factors are “factors derived from the health care production function, that interact to produce effective health care services” [10]. Access is hampered when individuals do not use services that could be beneficial to them on the demand side or when good quality effective care is not offered on the supply side, and both demand and supply sides are related [11]. Most studies that examine access to health care use frameworks that include the “4 As model” dimensions (that have supply and demand elements as described by Peters et al. [16]).

Individual and household factors that influence use of ANC include education, age, parity, socio-economic status, area of residence and knowledge about the characteristics of and need for medical treatment [13]. Cultural, religious and other social factors are among community factors that influence use of health services and these include autonomy, religion, social support and cultural beliefs [17–20].. A recent systematic review that examined provision and uptake of ANC emphasized the importance of traditional beliefs, which may consider pregnancy to be a normal event that does not require medical attention, in influencing women’s health seeking behavior in LMICs. Cultural beliefs and the role of others that influence women’s health seeking behavior in traditional societies are not captured with standard national household surveys.

Syria had made considerable progress in decreasing maternal and infant mortality before the conflict in 2011. Ninety six percent of women delivered with a skilled birth attendant, and 88% had at least one ANC with a skilled attendant. However, the number of women who had at least 4 ANC visits (according to the national guidelines following WHO recommendations at the time of the study) was much lower (64%) [21].

An earlier study by Mourtada et al. (2019) conducted secondary analyses of national health surveys which demonstrated wide regional variations in uptake, timing of first visit, number of visits to ANC and quality of ANC [22, 23]. Although women’s socio-demographic characteristics are important determinants of service use, Mourtada et al’ s analyses revealed that the regional disparities in ANC coverage and patterns of use remained even after controlling for women’s main demographics [20], suggesting that there are additional factors contributing to the regional disparities in ANC coverage and use patterns, which are likely related to service provision on the supply side and socio-cultural and community factors on the demand side.

The Syrian conflict caused destruction to many health facilities in the country consequently interrupting maternal health care for many women [24] and likely deepening divisions in care quality. Post-conflict reconstruction of health services offers a unique opportunity to address the issues, which contributed to the regional disparities in ANC coverage and patterns of use that existed pre-conflict. Efforts to rebuild the health system in Syria are likely to focus on addressing supply side factors that are related to governance, infrastructure, rebuilding the destroyed health facilities and retaining the health workers [25] with the overall aim of achieving universal health coverage. However, high coverage does not always translate into positive health outcomes when health facilities are underutilized [26]. Therefore, it is important to address the demand-side barriers to adequate uptake of ANC that contributed to the regional variations in ANC coverage and patterns of use that were present before the conflict.

## Methods

### Aims of the study

The main aim of this qualitative study is to explore barriers to women’s adequate uptake of ANC and regional differences in uptake. Findings are utilized to discuss potential low-cost interventions to increase women’s uptake of ANC focusing on addressing the demand side barriers, which are discussed within three dimensions of the “Right to Health” model (availability, accessibility (geographical and financial) and acceptability).

### Study design

This qualitative study employed semi-structured interviews and observation to explore factors affecting utilization and quality of ANC in Syria. It was carried out in 2010, shortly before the eruption of the conflict and was part of a larger mixed-methods study that analyzed data from two national surveys (Pan Arab Project for Family Health (PAPFAM) (1996–2001) and Multiple Indicator Cluster Survey (MICS) (2004–2006). In this paper, we present the perspectives of women in two governorates in Syria; Latakia where uptake of ANC was high and Aleppo where uptake of ANC was low to explore barriers to adequate uptake of ANC [21]. Inadequate uptake of ANC in this study is defined as: not seeking ANC, seeking care with providers who are not trained to provide ANC or discontinuing care.

#### Conceptual framework

**Fig. 1:**
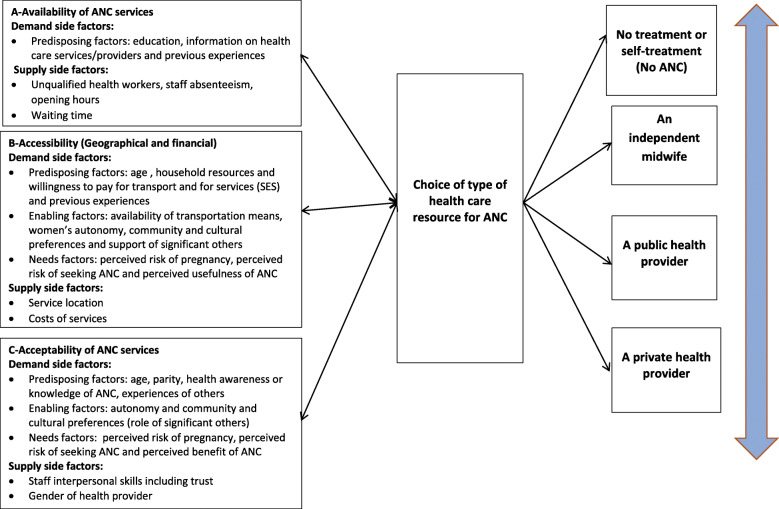
A modified conceptual framework based on three models for health care seeking behaviour

We have constructed a modified framework [23] (Fig. 1) that draws on a combination of two health care seeking behavior models: the Andersen and Newman Model that includes a wide range of women and health services related factors that demonstrate a strong influence on maternal health care service utilization in many settings; And Goods Pathway Model, which enables us to explore the role of the socio-cultural context, including the influence of significant others and the processes that guide women’s selection of their ANC health sources. Three elements of the “Right to Health” Model constitute the main construct of the conceptual model. Women’s choice of type of ANC service depends on the following dimensions of access: availability, accessibility (geographical and financial) and acceptability. We excluded quality as its definition (according to the “Rights to Health” framework focuses solely on the scientific and medical aspects of the service, which were examined in another study that focused on the provision of ANC content. In our model, we have classified the demand side and supply side factors that could be possible barriers to each dimension of access, following the classification suggested by Jacobs et al. [27]. For more details on the conceptual model, please refer to Appendix A (Additional File 1).

#### Study setting

Table 1 provides an overview of Aleppo and Latakia governorates (referred to as Aleppo and Latakia in the paper) including the population size in each governorate in 2010 at the time of the study. Aleppo is the largest and most densely inhabited Syrian governorate. Latakia is much smaller and its capital, Latakia city, is the main port city of Syria on the Mediterranean Sea.

**Table 1:** The general characteristics of Aleppo and Latakia governorates

	Aleppo	Latakia
**Location**	Northern Syria	Western Syria
**Land area**	1,850,000 ha	230,000 ha
**Population**	5,927,000	1,229,000
**Number of districts**	8	4
**Number of villages**	1453	432
**Religion**	90% are Muslims; the majority are Sunnis, mostly Arabs, followed by Kurds. The rest (10%) are Christians and Armenians	Mostly Alawites with some Sunni Muslims and Greek Orthodox Christians

#### Study participants

Participants included pregnant women aged 18–45 years living in Aleppo and Latakia attending ANC at different types of health facilities that provide ANC in Syria (private clinics/hospitals, public health centers/hospitals in urban and rural areas). We also included women who attended ANC with midwives who provided ANC independently at their own clinics in Aleppo (which was considered illegal as reported by health officials, as midwives in Syria are not trained to provide ANC independently, especially examining women using an ultrasound and prescribing ANC tests, without the supervision of a doctor) as well as women who did not attend ANC (Table 2).

**Table 2:** An overview of the interviewed women in Aleppo and Latakia governorates

	Women in Aleppo	Women in Latakia	Total
**Private clinics/hospitals**	4	7	11
**Public health centres/hospitals**	4	5	9
**Midwives clinics**	5	NA	5
**No ANC**	5	NA	5
**Total**	18	12	30

#### Sampling and recruitment

In each governorate, we employed purposive sampling to recruit the women from four randomly selected private clinics (from the list of private practices issued in 2009 by the Syrian Society of Obstetrics and Gynecology) and four randomly selected Public Health Centers (PHCs) (from the list issued in 2009 by the Ministry of Health). Please refer to Appendix B (Additional File 1) for a detailed description of the sampling process).

We contacted the doctor working at each health facility, explained the study and if they consented, we arranged to approach women at their health facilities. If doctors declined, we selected an alternative clinic/health center below them on the list. We approached pregnant women in the waiting areas at the health facilities independently of the health providers and emphasized that their decision to participate would not affect the health care they were receiving at this health facility. We gave them the option to be interviewed at a private room at the health facility after their appointment or at their homes at a time that is convenient for them. Women who preferred to be interviewed at their home shared their contact details. We contacted them the second day to arrange for their interview at home. None of the women we approached refused to participate.

To recruit women attending ANC with midwives who provide ANC independently, we obtained the phone numbers of midwives from the health officials in Aleppo who worked at the health directorate. We contacted the midwives, explained the study and approached women at the clinics of those who agreed to participate using the same method of recruitment as the other facilities. No midwives provided ANC independently in Latakia.

Women who did not attend ANC were recruited using the snowball technique of purposive sampling, with the help of women participating in our study. It was reflective of the regional differences in the pattern of ANC use that we were unable to locate and recruit women who did not attend ANC in Latakia. Our experience was confirmed by doctors and women in Latakia who confirmed that almost all women in Latakia attended ANC at least once. MICS survey data in 2006 shows that 97% of women in Latakia attended ANC and this percentage may have increased by the time we conducted our interviews in 2010 [21, 22].

We recruited 13 women attending ANC and 5 women not attending ANC in Aleppo, and 12 women attending ANC in Latakia (Table 2).

#### Data collection

Semi-structured audio-recorded interviews with pregnant women were carried out in both governorates. Semi-structured interviews allow the interviewees to talk more freely about their personal experiences and perceptions while also remaining focused on the topic [28].

The interviews were carried out in Arabic by the lead author as part of her PhD research. The interview guide covered the following topics: women’s practices regarding their current pregnancies (and the reasons for these practices); places where they sought care, frequency of care; women’s perceptions of the importance of ANC and certain elements of ANC. The guide was piloted with three women in each governorate and reviewed by the research panel.

We interviewed twelve women in Aleppo twice mainly because during the first interview these women were shy, and not always alone, which might have influenced their willingness to share information about their health seeking behavior. The interviews lasted between 30 and 45 min.

The lead author also conducted fifteen observation sessions, lasting approximately two hours each and recorded in fieldnotes at a range of health facilities in Aleppo and Latakia to validate the content of the interviews. The lead author observed the physical settings of the health facilities and the participants including women, doctors, staff and how they interacted in different settings.

The role of the researcher was known and agreed upon prior to the observations taking place [29, 30].

#### Data management and analysis

Interviews were transcribed verbatim and relevant quotes were translated into English. Data were analyzed by the lead author and codes were checked by a co-author using the Framework Method for health research, a modified version of content analysis developed for policy relevant research [31, 32]. Familiarization with the data and the identification of persistent and contrasting themes led to the construction of a detailed index where all data were annotated according to the thematic framework and charted. In the final stage (mapping and interpretation), charts were reviewed carefully, examining different perceptions and experiences looking for similarities and differences and attempting to find associations between themes to provide an explanation for the findings. The refined themes were discussed in relation to the three dimensions of access under the “Right to Health” framework (discussed earlier) (Availability, accessibility (geographical and financial) and acceptability) of ANC services focusing on the demand side barriers under each dimension.

## Results

### Characteristics of study participants

Demographic characteristics of participants are in (Additional File 2).

### Barriers to adequate uptake of ANC

Three dominant themes emerged throughout the semi-structured interviews that explained barriers to adequate uptake of ANC, particularly in Aleppo. These were related to women’s assessment of their own health status in pregnancy and reasoning about causes of ill health, women’s perception of risks of seeking ANC, and women’s appraisal of the value of different services and their treatment by providers.

#### Theme 1: Women’s assessment of their own health status in pregnancy and reasoning about the causes of ill health

Women in Aleppo who never attended ANC and those who attended ANC but did not follow up regularly assessed their own health status and the risks during pregnancy differently to women in Latakia. Interviews indicated that some women in Aleppo associated seeking care with being ill or experiencing complications during pregnancy or even with being infertile: *“We do not have the habit of seeking ANC here. I will go to the doctor only if I feel sick or if I bleed.” She added, “Some women in the village visit the doctor but those have fertility problems.” (Participant 4, rural Aleppo, never used ANC, first interview).*

While some women did seek ANC during their first pregnancy, those who had successful pregnancy and childbirth with no complications felt that there was little value in seeking care in subsequent pregnancies: *“I did not want to follow this pregnancy up, I tried the first pregnancy, and everything was normal.” (Participant 6, rural Aleppo, currently not attending ANC, first interview);* or chose to undertake a single visit during the pregnancy: *“I will visit the doctor but maybe I won’t register to follow up like the first time. The first baby is different. You are scared because you know nothing, this is why you follow up...” (Participant 18, Aleppo City, attended ANC, private clinic, second interview).*

By contrast, all interviewed multiparous women in Latakia reported attending ANC regularly, either with private or public doctors (Additional File 2).

Some of the interviewed women who experienced complications and miscarriages did not perceive ANC to be protective. Their explanations for their losses included having had a shock, a nightmare, or the evil eye: *“People in the village told me that it is the evil eye that caused my miscarriage, so my mother and my husband advised me to go to the sheikh to disable the evil eye.” (Participant 2, rural Aleppo, attended ANC, private clinic, one interview)*.

It is notable that women living in rural Aleppo did describe the value of visiting the local health center to get vaccinated against tetanus (free at point of access) in their interviews. Observations undertaken in the accessible rural Aleppo health centers showed however, that the doctor was not available on a regular basis and that the doctors practicing were mostly males which could have discouraged some women from seeking ANC given the traditional beliefs in those communities.

#### Theme 2: Women’s evaluation of the risks associated with seeking ANC services

Some women in Aleppo, mostly those living in rural areas, with little formal education and low-socioeconomic status, not only perceived there to be few benefits to attending ANC regularly, but further reported that seeking ANC might put their pregnancies at risk through the following: fear of hearing false bad news and fear of being asked to undergo a caesarean section. These perceptions were often founded on and expressed through stories from their immediate community (family members, friends and neighbors): *“I worry that the doctor might tell me some bad news that will stress me out. My cousin, who was nine months pregnant, was told by her doctor that the baby’s position was not right and she would need a caesarean. She was stressed out, and at the end, she had a normal delivery. This affected her and she was stressed out for a whole month (Participant 3, rural Aleppo, not attending ANC, second interview).”* Similarly, participant 1 explained: *“The doctor reassured me …*. *If he told me that there is something wrong, I would have been scared and I would have followed up with another doctor.” (Participant 1, Aleppo city, attending ANC, private clinic, second interview).*

Although some women in Latakia reported doubts about the usefulness of ANC or confidence in a doctor, they did not express similar fears that attending ANC could be the cause of harm: *“I monitored my previous pregnancy from the beginning and they did not discover that my baby was malformed until the end. Sometimes monitoring the pregnancy is useless unless both the doctor and the ultrasound are good.” (Participant 27, Latakia city, attended ANC, private clinic, one interview).*

Some women avoided seeking ANC with doctors due to the belief that most doctors performed unnecessary caesarean sections or hurried the ‘natural course’ of birth: *“The midwife does not deliver you unless you are totally ready whereas the doctor gives you needles or operates on you when your delivery is delayed” (Participant 5, rural Aleppo, did not attend ANC, first interview); “Midwives have more patience, the doctor will not wait. I heard several stories where doctors did not wait, they ask for a caesarean right away (Participant 16, Aleppo city, attended ANC with a midwife, first interview).”* Not only had these women avoided ANC because they did not perceive any benefits, these women perceived seeking care as a risky venture and avoided seeking ANC with certain providers such as doctors or avoided seeking ANC altogether to ‘protect’ their pregnancies.

Our observations confirmed that the roads to the health facilities in some of the areas in rural Aleppo and poor urban Aleppo were in a poor condition, public transport was irregular and the distances were greater compared to the journeys described by women in Latakia. This may have contributed to women’s perceptions of seeking ANC as risky.

#### Theme 3: Women’s appraisal of the value of different services and their treatment by providers

A central theme that emerged from the accounts of women who attended ANC was their preference for private care, which included care with a private doctor or a private independent midwife, over public health centers. This was due to the following reasons: lack of knowledge about services offered by public health centers (PHCs), lack of trust in PHC services and poor experiences of provider-patient interaction at PHCs.

Some women in Aleppo and in Latakia were not well informed about the services offered at PHCs by clinicians: *“The staff did not mention that I could do the tests at the health center” explained Participant 8 (Aleppo city, attended ANC, PHC, first interview).* Participant 21 who lived in Latakia city and attended ANC at a PHC explained: *“The service here is good. I have never heard of this health center before. My sister told me about it. I did not even know that they have a gynecologist and an ultrasound.” (one interview).*

Some women who were more familiar with the ANC services offered at PHCs however did not trust their quality, particularly, their ability to deal with or identify complications and preferred to visit a private doctor at the beginning of their pregnancy, only later following up the pregnancy at a PHC: *“I visited a private doctor at the beginning of my pregnancy. She reassured me that my pregnancy was fine. Therefore, I have decided to follow up at this health center [PHC].” (Participant 9, Aleppo city, attending ANC, second interview).*

A frequently reported experience in both governorates, but more commonly reported in Aleppo, was being mistreated by doctors and staff at PHC: “*they are cold, they do not make you feel comfortable, they do not understand what you want to tell them” (Participant 9, Aleppo city, attended ANC, PHC, first interview) and* “*I once visited a PHC but I lost my card and they did not agree to see me again, they refused to give me another card. They told me this is a lesson for you although I did not lose it on purpose.” (Participant 7, Aleppo city, not currently attending ANC, second interview)*. Impolite treatment of women by doctors and staff working in PHC in Aleppo was confirmed in observation sessions’ fieldnotes. For example, a doctor scolded a woman for not being able to tell them how severe her headache was saying: *“How difficult could it be to tell me?”* On another occasion, a woman was rudely scolded by a nurse at the health center for mistakenly entering the wrong room.

By contrast, women who attended care with independent midwives described their relationship with their midwives more positively: *“Although it takes half an hour to get here, I prefer to seek care with her. I like her as she makes me feel good every visit. Good attitude is the most important thing...especially during the last month of pregnancy.” (Participant 12, Aleppo city, attended ANC with a midwife, one interview); and* “*My midwife does not intimidate me and she never makes me feel I know nothing like doctors do.” (Participant 14, Aleppo city, attended ANC with a midwife, one interview).*

The majority of women in Latakia were generally satisfied with how the doctors and staff treated them: “*The doctor here is so nice and has sense of humor (she smiles). She makes me feel at ease. It is all about attitude...I have decided to follow-up here.” (Participant 29, rural Latakia, attends ANC, PHC, one interview).*

Although women in Aleppo expressed a preference for private clinics, their incapability to afford care limited their ability to attend ANC with a private doctor so women either discontinued ANC: *“The private doctor is too expensive, and the treatment at the health center is really bad, so we have to depend on God now, that’s all we have*” (*Participant 8, Aleppo city, attended ANC, PHC, second interview),* or attended care with independent midwives who charged lower fees: *“She is not expensive, I pay only 200 SP for a visit, and sometimes she does not take money, and when she refuses to take money I give her a present.” (Participant 15, Aleppo city, attended ANC with a midwife, second interview).*

## Discussion

The patterns of inadequate uptake of ANC as defined in this study (not seeking ANC, seeking care with providers who are not trained to provide ANC or discontinuing care) were prominent in Aleppo and the results generated insights into regional differences in ANC uptake. Overall, three dominant themes emerged, which explained different patterns of ANC seeking behavior among women in Aleppo and Latakia. The first theme focused on women’s assessment of their health status in pregnancy and reasoning about the causes of ill health. Such perceptions were related to experiencing complications, women’s parity level and their perception of the usefulness of ANC, and these were different for women in Latakia who described ANC as important regardless of their health status or parity level. The second theme described women’s evaluation of the potential risks associated with seeking ANC services, which included fear of hearing bad news and fear of being asked to undergo a cesarean section. Women in Latakia were less likely to have such concerns or weighed these up differently to women in Aleppo. The third theme examined women’s appraisal of the value of different services and their treatment by providers. Private providers were preferred to public services in both governorates and this was connected to the lack of knowledge about services provided at PHCs, lack of trust in services provided at PHCs and poor experiences of patient-provider interactions at PHCs. However, poor experiences with seeking ANC at PHCs were not reported to the same degree by women in Latakia.

We discuss our findings against the conceptual framework constructed for this study (Fig. 1) and under the three selected dimensions of access: availability, accessibility (geographical and financial) and acceptability focusing on the demand side barriers to accessing adequate ANC.

### Availability

Our results indicated that even when services were available, such as in the villages, where interviewed women did not seek ANC at all, there was low demand for them. A potential explanation for it could be that such women were often uneducated (Additional File 2) and consequently did not attempt to obtain information on such health facilities and the services they provide and were not empowered to do so (predisposing characteristics). On the other hand, the limited hours where doctors were present at such health facilities could also discourage women from attending ANC and may have contributed to the lower demand for such services.

### Accessibility (geographical and financial)

Our observations of the general settings confirmed that PHCs were often available and free of charge, however, geographical accessibility may have been an issue in some areas where road conditions were bad or regular transportation means were not available. As a result, accessibility (geographical and financial) of services could have been a challenge for vulnerable women in Aleppo with low autonomy, restricted access to financial resources and consequently limited mobility (enabling factors). Additionally, although it was not brought up during the interviews, women in traditional societies such as in Aleppo, are not allowed to leave the house unaccompanied (enabling factors), which makes seeking care even harder when services are not easily accessible. Therefore, even when women wanted to seek care despite their communities’ traditional norms that did not perceive ANC as important, they may have been faced with other traditional norms that did not favor women going out alone (enabling factors). Accessing ANC becomes even more challenging when women are obliged to pay for transportation to go to a PHC or pay for ANC at private clinics that are closer to where they live. Moreover, women often evaluated the risks entailed in accessing ANC services (perceived risks of ANC). The poor road conditions in Aleppo and the potential negative implications of travel on pregnancy could have discouraged women from seeking care. Travel related risks resulting from inadequate infrastructure, distance to health facilities and lack of transportation were also brought up as barriers to ANC uptake in several studies included in a review that looked at reasons why women do not use ANC in LMICs [20].

### Acceptability of ANC services

Several issues were raised by interviewed women, which may have influenced their acceptability of ANC services. These were mainly related to perceived risks of pregnancy, perceived benefits of ANC and perceived risks of ANC (needs factors). The majority of women who did not seek ANC were from rural Aleppo where services were often available and accessible but those women were willing to seek care only when they perceived their case as risky or when they had problems conceiving (perceived risks of pregnancy and perceived benefits of ANC). Women’s emic definitions of risk, which affected their perception of ANC benefits, differ from the etic or medical definitions which consider any pregnant woman at risk of developing complications. Whereas in those communities, pregnancy continues to be seen as a normal event that does not require regular medical follow-up, especially among multiparous women and women who did not experience any health complications or had a normal previous delivery.

Generally, women in traditional communities worry about the harmful effect of the evil eye on the unborn baby, which obliges many women to remain discrete about their pregnancy until their baby is born [33]. A number of women in rural and poor urban areas in Aleppo brought up the concept of “evil eye,” and a few were convinced that it was responsible for their previous miscarriages reflecting their lack of trust in ANC benefits (perceived benefits of ANC), possibly because they were given no alternative explanations incorporating biomedical explanation of illness (low awareness of ANC). Additionally, women’s fear of the harmful effect of the “evil eye” in these communities could have discouraged them or delayed them from seeking care, as seeking ANC would have been a clear announcement of their pregnancy (perceived risks of ANC). These beliefs did not disappear with time, possibly due to the isolation of these communities as well as the lack of interaction with other communities or the medical system, which may have contributed to increasing those women’s vulnerability. This was unlikely the case for Latakia, as its smaller size and its geographical location on the Mediterranean Sea may have permitted additional exposure to the outer world and consequently resulted in a wider exposure to endorsed information.

Women’s acceptability of ANC services affected their choice of type of ANC provider. For instance, women brought up issues such as fear of hearing false bad news and fear of undergoing a cesarean section when seeking care with doctors (perceived risks of ANC). Such fears clearly suggest the women’s lack of trust in biomedical care. Preferences for normal vaginal childbirth were brought by women in rural Bangladesh in a study that examined women and obstetricians’ attitudes towards cesarean delivery [34], however, the study revealed that women trusted their doctors’ decisions performing cesarean sections unlike the women in this study who decided to discontinue ANC or seek care with independent midwives.

Lack of trust in public health care providers and being maltreated at PHCs were two main reasons when why women decided to go to a private doctor, an independent midwife or discontinue ANC when they could not afford private care. Another potential explanation of why many women preferred care with midwives, which was not brought up by women, is the preference for female health providers, which is particularly important for traditional women in Aleppo (enabling factors). A previous study that included 500 women in Damascus, Syria, confirmed women’s preferences for females as birth attendants [35], so we assume that this might have been the case for women in Aleppo.

Poor staff attitude was among the main factors that prevent women from seeking ANC as demonstrated by a review that examined reasons why women seek ANC in LMICs [20]. Similarly, a recent study in Saudi Arabia demonstrated that the negative attitude of the staff and poor communication prevented women from attending ANC despite their awareness of its importance [36]. The same study also demonstrated the importance of the beliefs of others including family and community members and how they impaired women’s access to ANC [34].

These perceptions of ANC and acceptability of certain types of services were related to prevailing cultural norms and the influence of others (enabling factors), which resulted in not perceiving ANC as being beneficial and which could have prevented vulnerable women with low autonomy from adequate uptake of ANC. These findings are in line with findings from other studies included in a recent review of factors influencing women’s uptake of ANC [19]. Fourteen studies in the review confirmed the influence of traditional beliefs on shaping women’s ANC seeking behavior, which translated into avoiding biomedical care [19]. Additionally, 16 studies that were included in the review established that for many women, pregnancy is still considered as a normal event that does not require medical attention, especially in LMICs [19].

The results of the current study have important implications for consideration of introducing low-cost interventions that address the demand side barriers to adequate uptake of ANC. Although universal health coverage is important to achieve positive health outcomes, its effectiveness becomes limited when services are not used or under-used. Our study demonstrated that vulnerable women, such as women in Aleppo were less likely to demand for ANC services even when they were available and accessible. Future interventions intending to increase adequate uptake of ANC should aim at addressing the needs of vulnerable women and strengthening their capacity and capabilities to enable them to make their own decisions without having to depend on the opinions of others [26]. We recommend 3 promising and inter-related approaches to strengthen women’s competencies and eventually improve their uptake of ANC: education, empowerment using women’s groups and Mobile Health Technologies (mHealth) as potential platforms to deliver education and empowerment interventions (Table 3).

**Table 3:** Potential low-cost interventions to address the demand-side barriers to adequate uptake of antenatal care

	Dimensions of access		
	Availability	Accessibility (geographical and financial)	Acceptability
**Education**	Improves knowledge about available ANC services and type of services provided by different types of providers (private, public).	Improves knowledge about available health services that are closest to where women live and types of services provided at such health facilities.	Increases knowledge about benefits of ANC improving trust in biomedical care and reducing reliance on unreliable sources of information.
**Empowerment**	Encourages women to be more proactive and obtain information on health services.	Increases women’s autonomy to improve their access to resources that impact on their geographical and financial access to services.	Increases women’s autonomy to enable them to communicate more effectively with health providers, improving the patient-provider relationship and the acceptability of services.
**mHealth tools**	**A platform to deliver messages to promote behavior change and improve uptake of ANC**		

Increasing women’s access to education improves their status in the household and the community, improves their knowledge of ANC benefits and their knowledge about existing services and appropriate types of providers, thus increasing the likelihood of their adequate uptake of ANC. Another effective strategy in poor settings is empowerment using community mobilization through women’s groups that encourage women to discuss their needs and the barriers they face when seeking care and encourage them to seek care at health facilities consequently improving maternal and birth outcomes [26, 37–41]. When education is coupled with empowerment, it improves women’s accessibility to health services especially when women have an increased mobility as well as an increased access to financial resources. Additionally, education and empowerment improve women’s ability to communicate effectively with health care providers thus enabling them to raise their concerns regarding any negative news they may receive hence reducing the likelihood of being mistreated by health providers, and consequently improving their acceptability of ANC services.

The use of mobile phones was not widespread around the time of the study, especially among rural and poor women. Additionally, social platforms such as WhatsApp, which permit the free exchange of text and audio messages, were also not available. Given the high prevalence of mobile phones use these days [42], using Mobile Health technologies (mHealth) offers an effective and a low cost platform to promote behavior change and increase vulnerable women’s uptake of ANC. A systematic review on the effectiveness of using mHealth in improving usage of ANC demonstrated that there is a strong evidence that delivering text messages reminders and education to the phones of pregnant women can improve their uptake of ANC [43].

When designing interventions for vulnerable women with limited mobility and reduced access to financial resources, it is important to consider interventions with a delivery mechanism that meets their needs. In the context of Syria, mHealth could be an effective platform to deliver such interventions without the need for women to leave their homes or use transportation, which is particularly important in settings where insecurity remains to be a major concern. Such platforms can be used to send reminders thorough text messages and deliver educational text and audio recorded messages on the importance of seeking ANC, risks of not seeking care and on available PHCs that provide ANC that are close to where women live. Also, mHealth can be an effective platform to deliver empowerment interventions. Such low-cost interventions using existing resources are worth exploring in a low-resource setting such as in Syria.

One of the main strengths of this study is that, unlike many studies, it chose not to present a generalized picture of ‘Syrian women’, their health care seeking behavior and their choice of different types of ANC providers but examined regional differences and patterns of ANC use and beliefs. This approach was grounded in the use of quantitative data to inform the study design [22] and was particularly relevant for patterns of ANC use, which differed substantially by region. The study also employed observation sessions, which helped triangulate the findings of the interviews.

The sample included in this study is relatively small and not representative of all women in both governorates, but as in any qualitative research, the sample is not usually selected for statistical representativeness, as the main objective of the study is to understand social processes. We could have missed important information by not including more women who did not use ANC at all in Aleppo, women who did not seek ANC in Latakia and by not including more women in rural Latakia. However, quantitative analyses of earlier surveys demonstrated that both rural and urban women in Latakia seek adequate ANC.

Interviewing women at home made them feel more at ease, since they were at a familiar setting. However, since many of them lived in households with many family members, interviews were often interrupted, and other family members like mothers and mothers in –law who participated occasionally. The presence of other family members might have made women feel less comfortable discussing their health seeking behaviour and biased their answers. Thus, to avoid such bias, we interviewed women in Aleppo for a second time and for a longer period, which helped to build rapport, encouraging the women to feel more comfortable sharing information about their ANC practices.

Courtesy bias, where women tried to give answers in favour of their doctors might have occurred with women in Latakia, as most participants preferred to be interviewed at health facilitates. Consequently, being interviewed at health facilities where those women received ANC might have biased their answers towards favouring the services they have received at these particular health facilities and might have discouraged them from discussing the issues that they were not entirely satisfied with.

## Conclusions

We believe that our proposed interventions are likely to be effective in addressing the demand-side barriers to adequate uptake of ANC that existed pre-conflict and which were likely to have persisted post-conflict. Such interventions could be also explored to increase women’s uptake of other maternal health care services including family planning and use of modern contraception methods. Furthermore, given the physical destruction caused by the conflict, which led to a decrease in the number of health facilities and which instigated the internal displacement of a large number of people, future research is needed to explore the additional demand side barriers that internally displaced women are currently experiencing when seeking ANC in their new host communities and as a result of using new and probably strained health facilities. Crafting appropriate interventions to increase women’s uptake of ANC is likely to be more effective when it is contextual, pays attention to regional disparities in access and when it addresses the barriers that women are presently facing.

## Supplementary Information

**Additional file 1: Appendix A.** Conceptual Model. **Appendix B.** Sampling and recruitment.

**Additional file 2.** Demographic characteristics of women in Aleppo and Latakia governorates.

## Data Availability

The datasets used and/or analyzed during the current study are available from the corresponding author on reasonable request.

## Abbreviations

ANC: Antenatal Care
LMICs: Lower and Middle-Income Countries
MICS: Multiple Indicator Cluster Survey
PAPFAM: Pan Arab Project for Family Health
PHCs: Public Health Centers
WHO: World Health Organization
