# Supplementary material for: A qualitative study exploring barriers to adequate uptake of antenatal care in pre-conflict Syria: low cost interventions are needed to address disparities in antenatal care
Source: Contracept Reprod Med. 2021 Jun 1;6:17. doi: 10.1186/s40834-021-00156-7 (PMC8167987; doi:10.1186/s40834-021-00156-7)
Supplement: Supplementary file 1 — Additional file 1: Appendix A. Conceptual Model. Appendix B. Sampling and recruitment. [file 40834_2021_156_MOESM1_ESM.pdf]

Appendix A: Conceptual Model

We have constructed a modified framework (Fig 1) that draws on a combination of two health care seeking behavior models: the Andersen and Newman Model that includes a wide range of women and health services related factors that demonstrate a strong influence on maternal health care service utilization in many settings; And Goods Pathway Model, which enables us to explore the role of the socio-cultural context, including the influence of significant others and the processes that guide women's selection of their ANC health sources. The elements of the "Right to Health" Model constitute the main construct of the conceptual model. According to our conceptual framework, women's choice of type of ANC service depends on the following dimensions of access: availability, accessibility (geographical and financial) and acceptability. We excluded quality as its definition (according to the "Rights to Health" framework focuses solely on the scientific and medical aspects of the service, which were examined in another study that focused on the provision of ANC content. Each of the selected dimensions is determined by a set of supply and demand side factors that enable or construct access to adequate ANC. In our model, we have classified the demand side and supply side factors that could be possible barriers to each dimension of access, following the classification suggested by Jacobs et al (25).

An Overview of identified access barriers along supply and demand sides and four dimensions of access

**Supply-side barriers**	**Demand-side barriers**
Availability	
• Unqualified health workers, staff absenteeism, opening hours<br>• Waiting time	• Information on health care services/providers<br>• Education
Geographic accessibility	
• Service location	• Indirect costs to households (transport)<br>• Means of transportation Available
Economic accessibility or affordability	
• Costs of services including informal payments<br>• Private-public dual practices	• Household resources and willingness to pay (SES)
Acceptability	

• Staff interpersonal skills including trust and gender of health providers	• Low self-esteem and little assertiveness (Autonomy)<br>• Community and cultural preferences (role of significant others)<br>• Stigma<br>• Lack of health awareness (Education)

The demand side barriers were also labeled under the three set of factors suggested by Andersen and Newman; predisposing factors, enabling factors and needs factors fitting in variables that are based on both; literature review and their relevance to our study aims. All of the abovementioned factors influence women's choice of the health care source for ANC (self-treatment or no treatment (No ANC), independent midwife, public health centre or private clinic). The blue arrow in the model designates that women can move from one treatment source to another (during the course of pregnancy) because according to Goods' Pathway model, the choice of health care is a dynamic process influenced by both the role of significant others as well as the doctor-patient relationship.

Appendix B: Sampling and recruitment

Purposive sampling was used to recruit the women using updated lists of gynecologists practicing privately and publicly in Aleppo and Latakia (issued in 2009 by the Syrian Society of Obstetrics and Gynaecology office, and the Ministry of Health). For each of the governorates, we randomly selected four private clinics (from the list issued by the Syrian Society of Obstetrics and Gynecology) and four PHCs from the list issued by the Ministry of Health) using the random selection number method with a calculator by specifying the lower and upper limits of the number of health facilities in each list and generating a random number within those limits. We repeated the process until we obtained the desired number of the health facilities we wanted to recruit women from. We then contacted the doctor working at each health facility, explained the study and if they consented, we arranged an appointment to recruit women from their health facilities. If doctors declined to participate, we selected the clinic/health center below them on the list and contacted the doctor who worked there. To avoid doctors' coercion, we approached pregnant women in the waiting areas at the

health facilities where selected doctors worked. We explained to women that we are independent researchers, we explained the study purpose and procedure, and asked them if they would be interested to participate emphasizing that this will not affect the health care they are receiving at this health facility. To make women feel at ease, we gave them the option to be interviewed at a private room at the health facility after their appointment or at their homes at a time that is convenient for them. Women who preferred to be interviewed at their home shared their contact details. We contacted them the second day to arrange for their interview at home. None of the women we approached refused to participate.

To recruit women attending ANC with midwives who provide ANC independently, we obtained the phone numbers of midwives from the health officials in Aleppo who worked at the health directorate. We contacted the midwives, explained the study and recruited women from the clinics of those who agreed to participate using the same approach used when recruiting women attending ANC with doctors. There were no midwives that provided ANC independently in Latakia.

Women who did not attend ANC were recruited using the snowball technique, a type of purposive sampling, with the help of women participating in our study. We were unable to locate and recruit women who did not attend ANC in Latakia. Our experience was confirmed by doctors and women in Latakia who claimed that almost all women in Latakia attended ANC at least once. MICS survey data in 2006 also shows that 97% of women in Latakia attended ANC. This percentage might have increased in 2009-2010 around the time we conducted our interviews.

We recruited 13 women attending ANC and 5 women not attending ANC in Aleppo and 12 women attending ANC in Latakia. Twelve women in Aleppo were interviewed twice resulting in a total of 42 in-depth interviews.
