# Supplementary material for: A qualitative study exploring barriers to adequate uptake of antenatal care in pre-conflict Syria: low cost interventions are needed to address disparities in antenatal care
Source: Contracept Reprod Med. 2021 Jun 1;6:17. doi: 10.1186/s40834-021-00156-7 (PMC8167987; doi:10.1186/s40834-021-00156-7)
Supplement: Supplementary file 2 — Additional file 2. Demographic characteristics of women in Aleppo and Latakia governorates. [file 40834_2021_156_MOESM2_ESM.pdf]

Table 3: Demographic characteristics of women in Aleppo and Latakia governorates

Participants' number	Age	Parity	Education	Work status	Place of residence	ANC place
**1**	19	2nd pregnancy	Primary	Never worked	Rural Aleppo	Private hospital
**2**	30	7th pregnancy	Illiterate	Never worked	Rural Aleppo	Private doctor
**3**	19	1st pregnancy	Illiterate	Never worked	Rural Aleppo	No ANC
**4**	20	2nd pregnancy	Illiterate	Never worked	Rural Aleppo	No ANC
**5**	22	3rd pregnancy	Preparatory	Never worked	Rural Aleppo	No ANC
**6**	16	2nd pregnancy	Preparatory	Never worked	Rural Aleppo	No ANC
**7**	31	3rd pregnancy	Illiterate	Never worked	Aleppo city	No ANC
**8**	20	2nd pregnancy	Primary	Never worked	Aleppo city	Health centre
**9**	35	5th pregnancy	Primary	Never worked	Aleppo city	Health centre
**10**	20	1st pregnancy	Primary	Never worked	Aleppo city	Health centre
**11**	19	1st pregnancy	Primary	Never worked	Aleppo city	Public hospital
**12**	30	4th pregnancy	University	Currently working	Aleppo city	Midwife
**13**	20	2nd pregnancy	University	Currently working	Aleppo city	Midwife
**14**	29	6th pregnancy	Primary	Never worked	Aleppo city	Midwife
**15**	22	1st pregnancy	Secondary	Never worked	Aleppo city	Midwife
**16**	35	4th pregnancy	Primary	Never worked	Aleppo city	Midwife
**17**	35	3rd pregnancy	University	Currently working	Aleppo city	Private doctor
**18**	24	2nd pregnancy	Secondary	Never worked	Aleppo city	Private doctor
**19**	23	2nd pregnancy	Preparatory	Never worked	Rural Latakia	Health center
**20**	29	5th pregnancy	Preparatory	Never worked	Latakia city	Health centre
**21**	28	3rd pregnancy	preparatory	Never worked	Latakia city	Health centre
**22**	31	3rd pregnancy	University	Never worked	Latakia city	Health centre
**23**	27	3rd pregnancy	Preparatory	Never worked	Latakia city	Health centre
**24**	25	1st pregnancy	University	Never worked	Latakia city	Private doctor
**25**	24	1st pregnancy	University	Never worked	Latakia city	Private doctor
**26**	25	3rd pregnancy	University	Never worked	Latakia city	Private doctor
**27**	30	2nd pregnancy	University	Currently working	Latakia city	Private doctor
**28**	25	1st pregnancy	University	Currently working	Latakia city	Private doctor
**29**	38	4th pregnancy	Primary	Never worked	Rural Latakia	Private doctor
**30**	40	4th pregnancy	University	Never worked	Latakia city	Private doctor
